# In Vitro Mutagenic and Genotoxic Assessment of a Mixture of the Cyanotoxins Microcystin-LR and Cylindrospermopsin

**DOI:** 10.3390/toxins11060318

**Published:** 2019-06-04

**Authors:** Leticia Díez-Quijada, Ana I. Prieto, María Puerto, Ángeles Jos, Ana M. Cameán

**Affiliations:** Area of Toxicology, Faculty of Pharmacy, University of Sevilla, C/Profesor García González 2, 41012 Sevilla, Spain; ldiezquijada@us.es (L.D.-Q.); anaprieto@us.es (A.I.P.); mariapuerto@us.es (M.P.); camean@us.es (A.M.C.)

**Keywords:** genotoxicity, mutagenicity, Cylindrospermopsin, Microcystin-LR, mixture

## Abstract

The co-occurrence of various cyanobacterial toxins can potentially induce toxic effects different than those observed for single cyanotoxins, as interaction phenomena cannot be discarded. Moreover, mixtures are a more probable exposure scenario. However, toxicological information on the topic is still scarce. Taking into account the important role of mutagenicity and genotoxicity in the risk evaluation framework, the objective of this study was to assess the mutagenic and genotoxic potential of mixtures of two of the most relevant cyanotoxins, Microcystin-LR (MC-LR) and Cylindrospermopsin (CYN), using the battery of in vitro tests recommended by the European Food Safety Authority (EFSA) for food contaminants. Mixtures of 1:10 CYN/MC-LR (CYN concentration in the range 0.04–2.5 µg/mL) were used to perform the bacterial reverse-mutation assay (Ames test) in *Salmonella typhimurium*, the mammalian cell micronucleus (MN) test and the mouse lymphoma thymidine-kinase assay (MLA) on L5178YTk^±^ cells, while Caco-2 cells were used for the standard and enzyme-modified comet assays. The exposure periods ranged between 4 and 72 h depending on the assay. The genotoxicity of the mixture was observed only in the MN test with S9 metabolic fraction, similar to the results previously reported for CYN individually. These results indicate that cyanobacterial mixtures require a specific (geno)toxicity evaluation as their effects cannot be extrapolated from those of the individual cyanotoxins.

## 1. Introduction

Nowadays, a proliferation of cyanobacterial species can be seen globally because of water eutrophication and climate change, leading to an increasing occurrence of cyanotoxins [1,2,3]. Cyanotoxins are toxic secondary metabolites produced by various species of cyanobacteria, which involved an ample variety of compounds with different structural and physicochemical properties [4]. Humans may be exposed to cyanotoxins via different routes, but oral exposure by means of contaminated water and foods (fish, crops, vegetables and food supplements) is by far the most important [5,6]. Microcystins (MCs) and cylindrospermopsins (CYN) are among the most frequently investigated cyanotoxins due to their toxicity and extensive distribution.

MCs are cyclic heptapeptides and 246 variants were identified so far [7], with Microcystin-LR (MC-LR) as the reference congener. The liver is the main target organ in MC-LR toxicity because of its uptake into hepatocytes by the organic anion transport system [8]. MC-LR inhibits the protein serine/threonine phosphatases by covalent binding, especially PP1 and PP2. Thus, the proteins are hyperphosphorylated leading to the modification of cytoskeleton and disruption of actin filaments [9]. In addition, MCs induce oxidative stress [1,10], disrupt different enzymatic activities [11,12] and induce apoptosis [13]. MC-LR was classified as possible human carcinogen (Group 2B) by the International Agency of Research on Cancer (IARC) [14]. It can produce genotoxic effects in vitro and in vivo [15], although the mechanisms involved are not yet completely understood [16].

Cylindrospermopsins are guanidine alkaloid hepatotoxins with five known analogues [17]. Cylindrospermopsin (CYN) has zwitterionic characteristics, thus being highly water soluble and chemically stable at high temperatures and a wide range of pH [18,19]. For these reasons, humans can be more likely exposed to CYN than to other cyanotoxins as up to 90% of total CYN is presented in surrounding waters. Although the liver and kidney are target organs of CYN, other organs such us lungs, heart, thymus, stomach, spleen, intestinal tract, skin, nervous, immune, vascular and lymphatic systems could also be damaged [1,20,21,22].

The absorption mechanism of CYN is not totally elucidated, but it was shown that paracellular transport is involved in the intestinal uptake [1,23]. The main mechanisms of CYN toxicity is the irreversible inhibition of protein synthesis [24,25] and glutathione (GSH) depletion [26] related to the oxidative stress induced by CYN [27,28,29]. Moreover, the bioactivation of CYN by cytochrome P-450 plays an important role in its mechanism of toxicity [30]. CYN was shown to induce DNA fragmentation and DNA strands breaks [31,32,33,34,35,36,37,38]. However, it was not yet classified by its carcinogenic potential by the IARC.

Both cyanotoxins have been extensively studied individually, but there are very few studies that evaluate their combined effects, as indicated by the European Food Safety Authority (EFSA) [5]. The simultaneous occurrence of MCs and CYN was reported repeatedly [39,40]. They have different chemical structures and mechanisms of action, thus interaction phenomena such as synergism, antagonism or toxicity potentiation must be considered. Moreover, a risk assessment can be greatly influenced when diverging from individual toxin exposure to a multi-toxin exposure scenario. Gutiérrez-Praena et al. [41] found an antagonistic effect of CYN and MC-LR when investigating the cytotoxicity of binary mixtures in comparison to the individual toxins in HepG2 cells. Hercog et al. [42] observed a genotoxic potential of CYN/MC-LR mixtures comparable to that of CYN alone when using the micronucleus (MN) and comet assays in the same experimental model.

The exploration of the genotoxic potential of CYN/MC-LR applicable to food and feed safety assessment is of great current interest. EFSA has indicated the need for further data on the toxicity of cyanotoxins mixtures [5] following recommended genotoxicity testing strategies [43].

Thus, the purpose of this research was to assess the mutagenic and genotoxic potential of the CYN/MC-LR mixtures trough a complete battery of different in vitro tests. This battery included: (1) The bacterial reverse-mutation assay in five strains of *Salmonella typhimurium* (Ames test, OECD 471 [44]) which detects gene mutations in the absence and presence of the microsomal fraction S9; (2) the Micronucleus test (MN, OECD 487 [45]) on L5178Y Tk^+/−^ cells that detects clastogenic and aneugenic chromosome aberrations in the absence and presence of the microsomal fraction S9; (3) the standard and enzyme modified comet assays with restriction enzymes (Endonuclease III (Endo III) and Formamide pyrimidine glycosylase (FPG)) that detect DNA strand breaks and oxidative DNA damage in Caco-2 cells; (4) the mouse lymphoma thymidine-kinase assay (MLA, OECD 490 [46]) on L5178Y Tk^+/−^ cells to detect gene mutations in the timidine kinase (Tk) locus in the absence and presence of the microsomal fraction S9. The microsomal fraction S9 was used to assess if CYN/MC-LR genotoxicity is due to metabolic bioactivation of these toxins or due to the parent compounds.

## 2. Results

### 2.1. Ames Test

No signals of toxicity and/or test solutions instability were observed during the test performance. CYN/MC-LR mixtures did not induce changes in any of the *S. typhimurium* strains without S9 fraction (Table 1). On the contrary, a significant increase in the number of revertants per plate was observed with TA97A, TA102 and TA135 strains. However, a MI higher than 2 was not obtained in any of the assayed experimental conditions. Solvent controls (MetOH 2% and DMSO) did not induce statistical significant changes versus the negative controls.

### 2.2. Micronucleus Test

In the absence of S9 fraction, CYN/MC-LR mixtures did not increase the number of binucleated cells with MN in any of the concentration assayed (Table 2). However, a significant reduction of the cytokinesis-block proliferation index (CBPI) was observed at the highest concentration (1.35 µg/mL CYN + 13.5 µg/mL MC-LR). Positive controls for clastogens (MMC) and aneugens (colchicine) showed a significant increase in the frequency of binucleated cells with micronuclei (BNMN) (*p* < 0.01).

In the presence of S9 fraction, CYN/MC-LR induced an increase of BNMN (%) when compared to the negative control, but only at 1 µg/mL CYN + 10 µg/mL MC-LR this change was statistically significant (*p* < 0.01).

### 2.3. Mouse Lymphoma Thymidine-Kinase Assay (MLA)

Results of the MLA are shown in Table 3, Table 4 and Table 5. None of the evaluated CYN/MC-LR mixture concentrations induced a mutagenic response in the absence or presence of S9 fraction, neither after a short treatment (4 h) nor a long treatment (24 h). Concurrent vehicle control did not show changes in comparison to negative control (data not shown).

### 2.4. Standard and Enzyme-Modified Comet Assays

**Figure 1:**
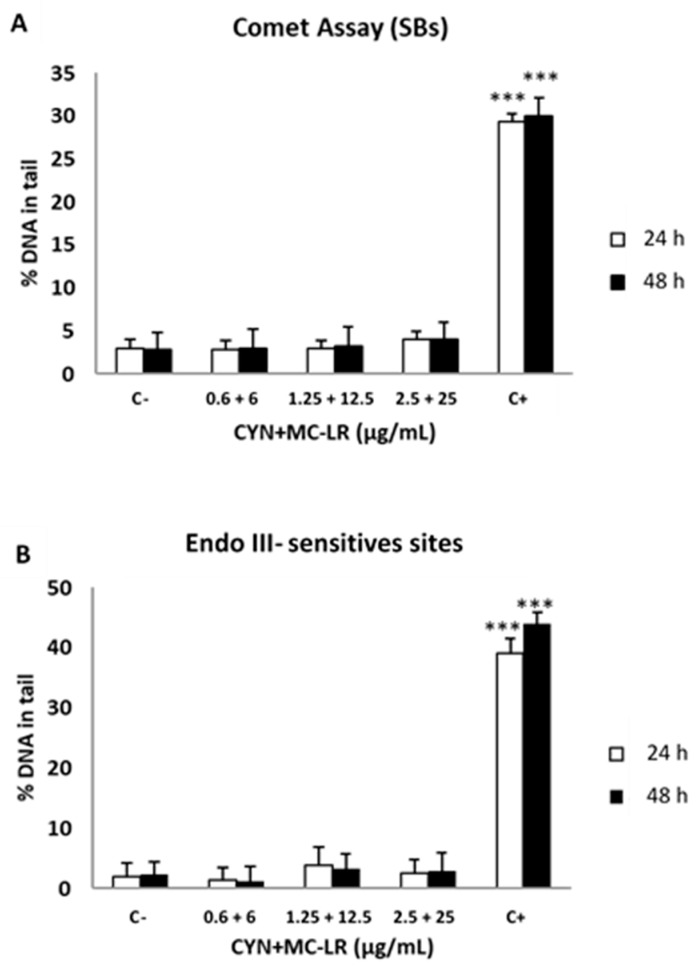

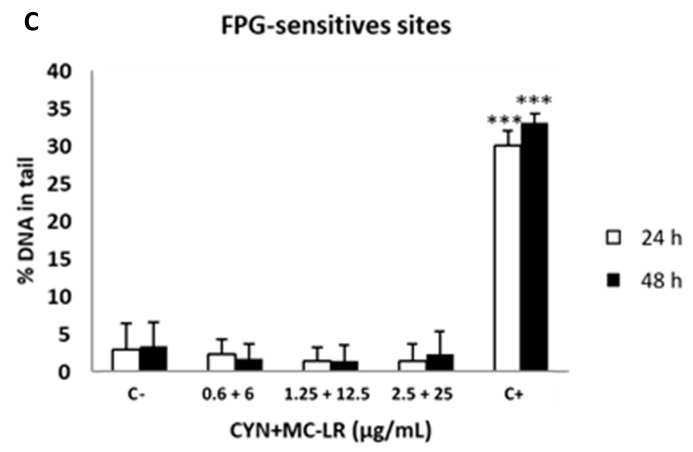
DNA damage in Caco-2 cells after exposure to CYN+MC-LR mixtures for 24 and 48 h. Results expressed as the formation of strand breaks (**a**) and oxidative DNA damage as Endo III-sensitive sites (**b**) and FPG-sensitive sites (**c**) (*n* = 3). The level of DNA strand-breaks (SBs), oxidized pyrimidines and oxidized purines are expressed as % DNA in tail. All values are expressed as mean ± SD. Negative control (C-): culture medium. Positive controls (C+): 100 μM H_2_O_2_ for the standard comet assay and Endo III-sensitive sites, and 2 μM of Ro 19-8022 photosensitizer with light irradiation for FPG-sensitive sites. *** *p* < 0.001.

Caco-2 cells exposure to CYN/MC-LR mixtures did not result in DNA strand breaks in the standard comet assay after 24 and 48 h (Figure 1a). In addition, an oxidative damage induced genotoxicity was not observed as the experiments performed with Endo III and FPG enzymes did not show a significant increase of % DNA in tail (Figure 1b,c). Results for the solvent control were similar to the negative control (data not shown) and only positive controls showed a significant (*p* < 0.001) genotoxicity.

## 3. Discussion

The data on the genotoxicity of a chemical is of key importance as it drives the type of human risk assessment to be performed. While a genotoxic chemical and health-based guidance value is usually set, for an unavoidable chemical, that is, a genotoxic carcinogen, the Margin of Exposure approach is usually applied [47]. For the generation and evaluation of data on genotoxic potential, the EFSA [43] recommends a step-wise approach for the generation and evaluation of data on genotoxic potential that begins with a basic battery of in vitro tests, including a bacterial reverse mutation assay and an in vitro MN assay. Moreover, further in vitro assays should be conducted in case of inconclusive, conflicting or equivocal results. The need for using several assays is justified as it is considered that there is no single mutagenicity test which can detect all kinds of potential human mutagens with 100% accuracy or prediction. This was shown to be true as mutagenesis itself is multifactorial [48].

Moreover, the genotoxicity evaluation of chemical mixtures is of great current interest and the EFSA has recently published a statement on the topic [49]. Thus, the Scientific Committee advocates for chemically fully defined mixtures, a component based approach, i.e., assessing all components individually using all suitable information including read across and quantitative structure–activity relationship (QSAR) considerations about their genotoxic potential, following the Scientific Committee guidance already mentioned [43]. In the present case, there are available data on CYN genotoxicity following EFSA recommendations [38], while MC-LR, was classified by the IARC in group 2B [14]. Moreover, the two single toxicity studies dealing with CYN/MC-LR mixtures have shown an antagonistic effect regarding cytotoxicity [41] and genotoxicity [42] in HepG2 cells. In addition, the genotoxicity of CYN/MC-LR mixtures has not been previously evaluated following a complete battery of in vitro tests, and a potential antagonic result for the mixture could affect the risk evaluation.

The first assay included in the basic battery was the Ames test. The mixture did not show a mutagenic response at the conditions tested, similar to previous results obtained for CYN [35]. In both cases, TA102 was one of the most responsive strains although the mutagenic indexes (MI) was always lower than 2. As CYN concentrations were similar in both studies, the results obtained suggest that MC-LR does not contribute to the genotoxicity of the mixture. This agrees with Sieroslawska [50] who found no effects in the Ames microplate format mutagenicity assay for pure MC-LR, pure CYN and neither for a mixture CYN/MC-LR/Anatoxin-a (1 µg/mL each).

A MN test is included in the basic battery to cover potential structural and numerical chromosome aberrations in addition to the Ames test. Chromosomal abnormalities, such as increased chromosomal breakage or chromosomal loss, are associated with enhanced risk of carcinogenesis and progression of neoplastic transformation [51]. In the case of the CYN/MC-LR mixture, an increase of MN was only observed with S9 fraction, similar to CYN in an individual exposure [38]. Moreover, single CYN showed this enhancement from lower concentrations (0.25 µg/mL) whereas the mixture showed this effect at 1 µg/mL CYN (+10 µg/mL MC-LR). This finding suggests that MC-LR ameliorates in this case the CYN response. However, in the scientific literature, there are contradictory data on the genotoxic potential of MC-LR by the MN assay. Thus, Abramsson-Zetterberg et al. [52] did not observe changes in vitro (in human lymphocytes, up to 2.0 mg extract of freeze-dried cyanobacteria per ml cell culture) and in vivo (in mice up to 55 µg/kg bw pure MC-LR by i.p. administration). On the contrary, Dias et al. [15] found that MC-LR treatment (5 and 20 μM) caused a significant induction in the MN frequency in kidney- (Vero-E6) and liver-derived (HepG2) cell lines and, interestingly, a similar positive effect was observed in mouse reticulocytes (37.5 μg MCLR/kg, i.p. route). Huang et al. [53] found that MC-LR induced a 1.6-fold increase in MN frequency in a human–hamster hybrid AL cell line after 30 days of exposure to 0.1 μg/mL (but no changes after 1 and 3 days of exposure). Regarding cyanobacterial mixtures, there is a single study that explored the MN induction of a CYN/MC-LR mixture and found that 0.5 µg/mL CYN + 1 µg/mL MC-LR induced a significant increase of MN in HepG2 cells [42].

Additional in vitro methods were applied (MLA and Comet assay), following the recommendations of [43], because the results obtained with the Ames test and the MN assay did not allow confirmation of the genotoxicity (or absence of genotoxicity) of the mixture.

The MLA results did not provide new evidence as no changes were observed at any of the conditions tested. Puerto et al. [38] also did not find a mutagenic response when single CYN exposure was evaluated. Zhan et al. [54] performed the TK gene mutation assay in the TK6 human lymphoblastoid cell line for MC-LR and found TK mutation in a concentration-dependent manner. The MLA is the most extensively used of the different in vitro mammalian gene-mutation assays [55]. Both MN assay and MLA are performed in the same experimental model, the L5178YTk^+/−^ cells, recommended in the Organization for Economic Co-operation and Development OECD guidelines. It seems that MN assay is more sensitive, or that the potential mutagenicity of the evaluated cyanotoxins is related mostly with chromosomal aberrations and to a lesser extent, with gene (point) mutations. However, the MLA detects intragenic events, mainly point mutations, and also loss of heterozygosity. This can result from the entire Tk gene loss, leading to karyotypically visible deletions and rearrangements of the Tk^+/−^ bearing chromosome [56]. These features make the MLA especially useful to evaluate the ability of chemicals to induce a broad variety of mutational events [57].

Similarly, the Comet assay also did not evidence DNA damage induced by the CYN/MC-LR mixture in any of the procedures performed, that is, the standard assay and the modified version to detect oxidative DNA-damage. CYN alone showed the same response in similar conditions: Experimental model, concentrations and times of exposure [38]. Other authors, however, have observed genotoxic effects for CYN in the Comet assay both in vitro [31,33,34] and in vivo [58,59]. MC-LR single exposure was also reported to induce DNA strand breaks by the comet assay in vitro [15,60,61,62] and in vivo [15]. There is a single study [42] that showed DNA strand breaks induction by cyanobacterial mixtures CYN/MC-LR in HepG2 cells after 24 h exposure, but to lesser degree than CYN. Once more, it seems that MC-LR ameliorates the genotoxicity induced by CYN.

Overall, it is difficult to derive any statement about the (geno)toxicity of CYN/MC-LR mixtures because the available studies in the scientific literature for the individual toxins mostly use different model systems and exposure concentrations. This is the first time that a thorough investigation using 4 different mutagenicity and genotoxicity assays has been performed for cyanobacterial mixtures and the results indicate that the mixture does not show a higher genotoxicity compared to CYN. However, taking into account that MC-LR was classified in the group 2B by the IARC due to its tumour promotion mechanism [14], caution is required when trying to elucidate its role in the mixture toxicity.

As Zouaoui et al. [63] highlighted, the type of interactions among toxins could be related with the different chemical structures and properties, and the competition or not, for the same cell receptor. It is, therefore, required to explore the cyanotoxins mechanisms of action when they are alone or in mixtures. In this case, the investigated cyanotoxins showed different toxicity mechanisms but also share others, such as the oxidative stress induction. Thus, Gutiérrez-Praena et al. [41] suggested that the depletion of GSH could be related with the antagonistic response as it could decrease the uptake ratio of CYN. Other authors such as Hercog et al. [42] pointed out to their different kinetics as MC-LR and CYN are detoxified and toxified, respectively, after [30,64] and also to the compromise of DNA repair mechanisms induced by MC-LR [65]. In any case, further studies would be required to fully understand the mechanisms involved in the toxicity of mixtures. Moreover, despite using the battery proposed by EFSA [43], considering the results obtained (positive effects only in one of the four tests performed) and the limitations of in vitro genotoxicity tests to predict the in vivo situation suggested by Nesslany [66], the further step would be to assess in vivo the genotoxicity of cyanobacterial mixtures.

## 4. Conclusions

The in vitro mutagenicity and genotoxicity showed by CYN/MC-LR mixtures do not differ substantially from that observed for CYN tested individually. This effect was evident only when S9 fraction was used, indicating the relevance of CYN on the mixture toxicity at the conditions tested. The increased knowledge of cyanotoxins mixture genotoxic potential would contribute to perform more realistic risk evaluations.

## 5. Materials and Methods

### 5.1. Chemicals and Reagents

Cylindrospermopsin (95% purity) and Microcystin-LR (99% purity) standards were provided by Alexis Corporation (Lausen, Switzerland). Chemicals for different assays were supplied by Gibco (Biomol, Sevilla, Spain), Sigma -Aldrich (Madrid, Spain), C-Viral S.L. (Sevilla, Spain) and Moltox (Trinova, Biochem, Germany).

### 5.2. Cells and Culture Conditions

Five *Salmonella typhimurium* histidine-auxotrophic strains TA97A, TA98, TA100, TA102 and TA1535 were used for the Ames test. L5178Y Tk^+/−^ mouse lymphoma cells used for the MN test and MLA were originally provided by Dr. Oliver Gillardeux (Safoni-Synthélabo, Paris, France). Caco-2 cell line, used for standard and enzyme-modified comet assays, come from a human colon adenocarcinoma (ATCC© HTB-37). L5178Y Tk^+/−^ cells and Caco-2 cells were maintained in an incubator with 5% CO_2_ and 95% relative humidity at 37 °C.

### 5.3. Test Solutions

Stock solution of CYN (1000 µg/mL) and MC-LR (4000 µg/mL) were prepared in milliQ sterile water and water: MeOH, respectively and stored at less than 4 °C. The exposure concentration solutions were prepared by dilution in sterile MilliQ water (Ames test), RPMI 1640 medium (MN and MLA assays) or MEM medium (standard and enzyme-modified comet assays). Test concentrations were selected individually for every test as they need to fulfil toxicity criteria in each of the experimental models used. The selected concentrations of MC-LR were 10 times higher than that of CYN since MC-LR is normally more abundant in nature [1,2,67].

### 5.4. Bacterial Reverse Mutation Test (Ames Test)

The Ames test was performed following the OECD Guideline 471 [44] and Maron et al. [68] with minor modifications as follows. Five *Salmonella typhimurium* histidine-auxotrophic strains (TA97, TA98, TA100, TA102 and TA1535) obtained from TRINOVA BIOCHEM GmbH (Germany) were cultured following the provider instructions. The mutagenic activity of CYN/MC-LR mixtures was assessed in the absence and presence of the external metabolic activation system from rat livers (S9 fraction). Each experiment was conducted with five growing concentrations of CYN/MC-LR mixtures (0.125–2 µg/mL CYN and 1.25–20 µg/mL MC-LR) selected according to the results obtained by Puerto et al. [38] when CYN mutagenicity was assessed by the Ames test. Also, a negative control (distilled sterile water), solvent controls (MeOH and DMSO) and a positive control for each strain in accordance with the presence or absence of S9 fraction were included. Nine-aminoacridine (50 µg/plate) was the positive control for TA97A without S9 fraction; 2-Nitrofluorene (2-NF) (0.1 µg/plate) for TA98; sodium azide (NaN_3_) (1 µg/plate) for TA100 and TA1535; and mitomycin C (MMC) (2.5 µg/plate) for TA102. The positive control in the presence of S9 fraction was 2-aminofluorene (2-AF) (20 µg/plate) for all strains. At least 3 independent experiments were performed using triplicate plates for each test concentration. Results are expressed as revertant colonies and mutagenic indexes (MI).

### 5.5. Micronucleus Test (MN)

The MN test was carried out following the OECD guideline 487 [45]. L5178Y Tk^+/−^ cells were seeded at a density of 2.0 × 10^5^ cell/mL and exposed to five different concentrations of CYN/MC-LR mixture (0.084–1.35 µg/mL CYN and 0.84–13.5 µg/mL MC-LR in the absence of S9 fraction for 24 h, and 0.125–2 µg/mL CYN and 1.25–20 µg/mL MC-LR for 4 h in the presence of S9 fraction). These concentrations were selected taking into account previous results obtained in cytotoxicity assays and carried out according to the OECD Guideline 487 [45]. The RPMI medium was used as negative control; MeOH as vehicle control; and 0.0625 µg/mL MMC and 0.0125 colchicine (without S9 fraction) and 8 µg/mL cyclophosphamide (CP) (with S9 fraction) as positive controls. Cells were exposed to CYN/MC-LR mixtures (4 or 24 h, with and without S9 mix, respectively), then exposed to cythochalasin B (Cyt-B) (6 µg/mL) for 20 h to block cytokinesis and obtain binucleated cells. Afterward, cells were exposed to a hypotonic treatment with KCl and fixed. Subsequently, cells were dripped on slides and stained with Giemsa 10%. Quantification of binucleated cells with micronuclei (BNMN) and cytokinesis-block proliferation index (CBPI) were carried out following the OECD 487 guideline [45] by analysing at least 2000 binucleated cells/concentration.

### 5.6. Mouse Lymphoma Thymidine-Kinase Assay (MLA)

The MLA assay was performed in agreement to OECD Guideline 490 [46] and Maisanaba et al. [69]. Each experiment includes a negative control (fresh media), a solvent control (MeOH), a positive control (methylmetanosulfonate, MMS 10 µg/mL in absence of S9 fraction and cyclophosphamide, CP 3 µg/mL in presence of S9 fraction), five concentrations of CYN/MC-LR mixture in the absence of S9 fraction for 4 and 24 h assays (0.04–0.67 µg/mL CYN and 0.4–6.7 µg/mL MC-LR) and six concentrations in the presence of S9 fraction for 4 h assay (0.04–1.35 µg/mL CYN and 0.4–13.5 µg/mL MC-LR). These concentrations were selected in accordance with previous tests performed to define the cytotoxicity of CYN/MC-LR mixtures by the relative total growth (RTG) after 4 and 24 h of treatment without S9 fraction. According to the ICH Expert Working Group [70], the highest concentration chosen for the mutagenicity test must be higher than 10–20% of RTG. RTG values were employed to determine the acceptability of the toxicity at each concentration. Cells were seeded at 10^4^ cells/mL in 96-well plates (two replicates per experimental group) to assess the viability and mutagenicity. The mutation analysis cells were exposed to 4 µg/mL trifluorothymidine (TFT), and both the viability plates and the mutagenicity plates were incubated at 37 °C and 5% CO_2_ for 12 days. Afterwards, viable colonies and TFT mutation colonies were counted. Thiazolyl blue tetrazolium (MTT) (2.5 mg/mL) was added to wells to facilitate the counting of mutant colonies, and the plates were incubated for 4 h. According to Honma et al. [71], the size of the colonies were described as small (less than 1/3 of well diameter) or large (higher than 1/3 of well diameter) colonies. Moreover, the induced mutant frequency (IMF) was also analyzed.

### 5.7. Standard and Enzyme-Modified Comet Assay

The standard comet assay was carried out to evaluate genotoxicity, and a modified version of this assay with endonuclease III (Endo III) and formamidopyrimidine (FPG), which recognise oxidized pyrimidines and purines, was performed to determine oxidative DNA damage, respectively.

The standard and enzyme-modified comet assays were carried out to assess the genotoxicity of CYN/MC-LR mixtures, as previously described by Collins et al. [72] and Llana-Ruiz-Cabello et al. [73]. Caco-2 cell line was selected as cyanotoxins are food contaminants and it is a commonly used enterocytic model in toxicological studies [74,75,76,77]. Cells were seeded at 3.5 × 10^5^ cells/mL into 24-well tissue culture plates and treated with increasing concentrations of CYN/MC-LR mixtures (0.6–2.5 µg/mL CYN and 6–25 µg/mL MC-LR) for 24 h and 48 h, according to the value obtained in the most sensitive cytotoxicity endpoint assayed [76]. Cells were treated with a negative control (medium) and a positive control (H_2_O_2_ 100 μM) for standard comet assay and Endo III sensitives sites and Ro 19-8022 (2 µM) for FPG-sensitive sites. After exposure time, cells were washed, trypsinized and re-suspended in phosphate buffer saline (PBS) at 2.5 × 10^5^ cell/mL. Cells suspensions were mixed with 1% (*w*/*v*) low-melting-agarose in PBS and placed on agarose precoated glass slides. Afterwards, lysis, incubation with Endo III and FPG (in the case of modified comet assay), denaturing, electrophoresis, neutralization, washing, fixation, dying, staining with SYBR Gold and quantification of nuclei were performed.

Olympus BX61 (fluorescence microscope) with the comet assay IV software (Perceptive Instruments, UK) available at the Microscopy Service of the University of Seville (CITIUS) was used to score the cells. The results were expressed as mean % DNA in tail respect to the negative control group. The % DNA in tail represents the amount of DNA breakage. Both types of comet assays (standard and modified) were performed in at least three independent experiments and using a triplicate/experiment.

### 5.8. Statistical Analysis

The statistical analysis was performed with Graph-Pad InStat software (Graph-Pad Software Inc., La Jolla, CA, USA). The non-parametric Wilcoxon matched-pairs signed-rank test was employed to compare the exposed samples with the negative control. Differences were considered significant at * *p* < 0.05, ** *p* < 0.01 and *** *p* < 0.001, respectively.

## Figures and Tables

**Table 1:** Effect of CYN-MC-LR mixtures on the Ames test in three independent experiments by triplicate. Data are given as mean ± SD revertants/plate. * *p* < 0.05. ** *p* < 0.01 in comparison to negative control.

Concentration (µg/mL)		TA97A				TA98				TA100				TA102				TA1535			
		−S9	MI	+S9	MI	−S9	MI	+S9	MI	−S9	MI	+S9	MI	−S9	MI	+S9	MI	−S9	MI	+S9	MI
Pure CYN-MC-LR mixture	Negative controls	231 ± 42	-	244 ± 5	-	21 ± 2	-	24 ± 9	-	117 ± 25	-	135 ± 14	-	215 ± 12	-	292 ± 11	-	293 ± 23	-	273 ± 33	-
	0.125–1.25	297 ± 37	1.4	319 ± 51	1.3	19 ± 2	0.9	18 ± 8	0.8	136 ± 40	1.2	153 ± 21	1.1	230 ± 36	1.1	440 ± 29 **	1.5	327 ± 25	1.1	376 ± 54 *	1.3
	0.25–2.5	165 ± 28	0.8	334 ± 49 **	1.4	20 ± 1	1.0	17 ± 7	0.7	144 ± 12	1.2	166 ± 24	1.2	217 ± 29	1.0	380 ± 33 **	1.3	311 ± 10	1.1	411 ± 54 **	1.4
	0.5–5	213 ± 15	1.0	290 ± 58	1.2	26 ± 9	1.3	20 ± 10	0.9	154 ± 13	1.3	143 ± 19	1.1	251 ± 17	1.2	296 ± 18	10.	309 ± 42	1.1	336 ± 18 *	1.1
	1–10	168 ± 10	0.8	234 ± 43	1.0	21 ± 2	1.0	19 ± 9	1.0	146 ± 18	1.2	130 ± 10	1.0	134 ± 12	0.6	383 ± 44 **	1.3	250 ± 43	0.9	464 ± 44 **	1.6
	2–20	205 ± 31	1.0	295 ± 25	1.2	19 ± 5	0.9	25 ± 8	1.0	104 ± 31	0.9	143 ± 19	1.1	151 ± 1	0.7	397 ± 32 **	1.4	276 ± 15	0.9	476 ± 52 **	1.6
	Positive controls	613 ± 66 **	2.9	527 ± 19 **	2.2	883 ± 55 **	42.0	960 ± 53 **	40.9	816 ± 11 **	7.0	583 ± 39 **	4.3	950 ± 118 **	4.4	671 ± 22 **	2.3	833 ± 25 **	2.8	659 ± 39 **	2.2
MeOH 2%		176 ± 25	0.8	316 ± 32	1.3	17 ± 5	0.8	25 ± 13	1.1	92 ± 13	0.8	87 ± 29	0.6	192 ± 8	0.8	280 ± 12	0.6	313 ± 9	1.1	233 ± 35	0.9
DMSO		209 ± 66	1.3	184 ± 38	0.8	25 ± 2	1.2	30 ± 6	1.3	115 ± 5	1.0	113 ± 17	0.8	250 ± 65	1.2	231 ± 35	0.8	342 ± 63	1.2	298 ± 16	1.1

Negative control: Milli Q water. Control solvent: MeOH 2% and DMSO. Positive controls without S9 for TA97A: 9-aminoacridine (50 µg/plate), TA98: 2-nitrofluorene (0.1 µg/plate), TA100 and TA1535: NaN3 (1.5 µg/plate) and TA102: mytomicin C (2.5 µg/plate). Positive control for all strains with S9: 2-aminofluorene (20 µg/plate).

**Table 2:** Percentage of binucleated cells with micronuclei (BNMN) and cytokinesis-block proliferation index (CBPI) in cultured mouse lymphoma cells L5178YTk^+/−^ exposed to CYN+MC-LR mixture (*n* = 3). The genotoxicity assay was performed in the absence and presence of the metabolic fraction S9. The values are expressed as mean ± SD. ** *p* < 0.01, *** *p* < 0.001 in comparison to negative control group values.

Experimental Group	Absence of S9				Presence of S9			
	Exposure Time (h)	Concentrations (µg/mL)	BNMN (%) ± SD	CBPI ± SD	Exposure Time (h)	Concentrations (µg/mL)	BNMN (%) ± SD	CBPI ± SD
Negative control	24	-	2.3 ± 0.5	1.9 ± 0.1	4	-	2.5 ± 1.0	1.8 ± 0.1
Positive control	24	Mitomycin C 0.0625	10.5 ± 4.1 ***	1.5 ± 0.1 ***	4	Cyclophosfamide 8	8.3 ± 1.9 **	1.8 ± 0.1
		Colchicine 0.0125	9.6 ± 1.7 ***	1.8 ± 0.0				
CYN+MC-LR	24	0.084–0.84	1.8 ± 1.5	1.9 ± 0.0	4	0.125–1.25	4.8 ± 2.6	1.8 ± 0.1
	24	0.168–1.68	2.3 ± 1.0	1.9 ± 0.0	4	0.250–2.5	4.0 ± 1.4	1.8 ± 0.1
	24	0.336–3.36	2.5 ± 0.6	1.8 ± 0.0	4	0.5–5	5.8 ± 1.5	1.8 ± 0.1
	24	0.672–6.72	1.3 ± 0.5	1.7 ± 0.1	4	1–10	8.8 ± 4.2 **	1.8 ± 0.1
	24	1.35–13.5	0.8 ± 1.0	1.3 ± 0.3 ***	4	2–20	4.8 ± 0.5	1.8 ± 0.1

Clastogen and aneugen positive controls: mitomicyn C (0.0625 µg/mL) and colchicine (0.0125 µg/mL), respectively.

**Table 3:** Toxicity and mutagenicity of CYN/MC-LR in L5178YTk^+/−^ cells after 4 h without S9 fraction by the mouse lymphoma thymidine-kinase assay (MLA) (*n* = 2). ^a^: Total mutant frequency divided into small/large (S/L) colony mutant frequencies. The induced mutant frequency (IMF) was determined according to the formula IMF = MF-SMF, where MF is the test culture mutant frequency and SMF is the spontaneous mutant frequency. *** *p* < 0.001.

Concentration (µg/mL)	Relative Total Growth		Percent Plating Efficiency		Mutant Frequency (× 10^−6^)		MF (S/L) ^a^		IMF (MF-SMF) (× 10^−6^)	
	Experiment 1	Experiment 2	Experiment 1	Experiment 2	Experiment 1	Experiment 2	Experiment 1	Experiment 2	Experiment 1	Experiment 2
0	100	100	91	124	107	152	51/56	33/41	-	-
0.04 CYN-0.4 MC	77	90	98	98	126	143	95/48	86/57	56	70
0.08 CYN-0.8 MC	98	100	93	70	202	157	111/91	102/55	95	83
0.16 CYN-1.6 MC	82	86	102	82	71	162	44/27	100/62	−14.4	89
0.33 CYN-3.3 MC	64	72	98	91	165	150	84/81	80/70	58	76
0.67 CYN-6.7 MC	57	58	95	88	174	144	106/68	60/84	67	71
MMS (10 µg/mL)	46	70	69	82	728 ***	738 ***	407/321	424/314	621	664

Positive controls: methylmethanesulfonate, MMS 10 μg/mL without S9 fraction and cyclophosphamide, CP 3 μg/mL with S9 fraction.

**Table 4:** Toxicity and mutagenicity of CYN/MC-LR in L5178YTk^+/−^ cells after 4 h with S9 fraction by the mouse lymphoma thymidine-kinase assay (MLA) (*n* = 2). ^a^: Total mutant frequency divided into small/large (S/L) colony mutant frequencies. The induced mutant frequency (IMF) was determined according to the formula IMF = MF-SMF, where MF is the test culture mutant frequency and SMF is the spontaneous mutant frequency. *** *p* < 0.001.

Concentration (µg/mL)	Relative Total Growth		Percent Plating Efficiency		Mutant Frequency (× 10^−6^)		MF (S/L) ^a^		IMF (MF-SMF) (× 10^−6^)	
	Experiment 1	Experiment 2	Experiment 1	Experiment 2	Experiment 1	Experiment 2	Experiment 1	Experiment 2	Experiment 1	Experiment 2
0	100	100	93	102	155	146	96/59	82/64	-	-
0.04 CYN-0.4 MC	96	84	82	84	94	100	43/51	42/58	−61	−46
0.08 CYN-0.8 MC	82	72	91	91	95	95	50/45	50/45	−60	−51
0.16 CYN-1.6 MC	58	51	95	100	98	95	48/50	49/47	−57	−51
0.33 CYN-3.3 MC	58	56	102	100	98	105	56/43	60/45	−57	−41
0.67 CYN-6.7 MC	26	31	118	113	120	132	62/58	77/55	−35	−14
1.35 CYN-13.5 MC	16	16	130	116	70	91	29/41	38/53	−85	−55
CP (3 µg/mL)	99	81	65	73	480 ***	433 ***	228/252	213/220	325	286

Positive controls: methylmethanesulfonate, MMS 10 μg/mL without S9 fraction and cyclophosphamide, CP 3 μg/mL with S9 fraction.

**Table 5:** Toxicity and mutagenicity of CYN/MC-LR in L5178YTk^+/−^ cells after 24 h without S9 fraction by the mouse lymphoma thymidine-kinase assay (MLA) (*n* = 2). ^a^: Total mutant frequency divided into small/large (S/L) colony mutant frequencies. The induced mutant frequency (IMF) was determined according to the formula IMF = MF-SMF, where MF is the test culture mutant frequency and SMF is the spontaneous mutant frequency. *** *p* < 0.001.

Concentration (µg/mL)	Relative Total Growth		Percent Plating Efficiency		Mutant Frequency (× 10^−6^)		MF (S/L) ^a^		IMF (MF-SMF) (× 10^−6^)	
	Experiment 1	Experiment 2	Experiment 1	Experiment 2	Experiment 1	Experiment 2	Experiment 1	Experiment 2	Experiment 1	Experiment 2
0	100	100	113	124	170	170	106/72	87/92	-	-
0.04 CYN-0.4 MC	103	115	90	87	107	78.9	62/45	48/30	−71	−100
0.08 CYN-0.8 MC	91	102	102	93	121	124	50/71	72/52	−57	−55
0.16 CYN-1.6 MC	79	96	76	108	143	100	81/66	56/44	−35	−79
0.33 CYN-3.3 MC	71	74	116	104	115	168	64/51	109/59	−63	−12
0.67 CYN-6.7 MC	39	39	127	104	113	195	74/39	77/118	−66	16
MMS (10 µg/mL)	52	66	35	34	778 ***	897 ***	370/408	459/438	599	718

Positive controls: methylmethanesulfonate, MMS 10 μg/mL without S9 fraction and cyclophosphamide, CP 3 μg/mL with S9 fraction.
